# Specificity of Signal-Binding via Non-AHL LuxR-Type Receptors

**DOI:** 10.1371/journal.pone.0124093

**Published:** 2015-04-29

**Authors:** Sophie Brameyer, Ralf Heermann

**Affiliations:** Biozentrum, Bereich Mikrobiologie, Ludwig-Maximilians-Universität München, Martinsried/München, Germany; University of Oklahoma Health Sciences Center, UNITED STATES

## Abstract

Quorum sensing is a typical communication system among Gram-negative bacteria used to control group-coordinated behavior via small diffusible molecules dependent on cell number. The key components of a quorum sensing system are a LuxI-type synthase, producing acyl-homoserine lactones (AHLs) as signaling molecules, and a LuxR-type receptor that detects AHLs to control expression of specific target genes. Six conserved amino acids are present in the signal-binding domain of AHL-sensing LuxR-type proteins, which are important for ligand-binding and -specificity as well as shaping the ligand-binding pocket. However, many proteobacteria possess LuxR-type regulators without a cognate LuxI synthase, referred to as LuxR solos. The two LuxR solos PluR and PauR from *Photorhabdus luminescens* and *Photorhabdus asymbiotica*, respectively, do not sense AHLs. Instead PluR and PauR sense alpha-pyrones and dialkylresorcinols, respectively, and are part of cell-cell communication systems contributing to the overall virulence of these *Photorhabdus* species. However, PluR and PauR both harbor substitutions in the conserved amino acid motif compared to that in AHL sensors, which appeared to be important for binding the corresponding signaling molecules. Here we analyze the role of the conserved amino acids in the signal-binding domain of these two non-AHL LuxR-type receptors for their role in signal perception. Our studies reveal that the conserved amino acid motif alone is essential but not solely responsible for ligand-binding.

## Introduction

Bacteria constantly need to monitor changing environments and hosts to adapt accordingly the bacterial group-behavior. Typically, this process of cell-cell communication is mediated via quorum sensing (QS) systems among proteobacteria. Thereby, the bacterial behavior is controlled dependent on the population size by communication via small diffusible molecules. The basic molecular QS system of Gram-negative bacteria consists of a LuxI-like autoinducer synthase and a LuxR-type receptor that detects the signaling molecule to control expression of specific target genes [1]. Typically, Gram-negative bacteria use acyl-homoserine lactones (AHLs) for communication, which are constantly synthesized by LuxI at a basal level, and sensed by the cognate LuxR-like receptor when exceeding a threshold concentration. However, several LuxR-type proteins show the modular domain structure of QS LuxR family members, but do not possess a cognate LuxI synthase. These LuxR proteins are referred to as LuxR orphans [2] or solos [3]. Strikingly, the three enteric and insect pathogenic *Photorhabdus* species, *P*. *luminescens*, *P*. *temperata* and *P*. *asymbiotica*, harbor an exceptional high number of LuxR solos [4], however the signals sensed by these LuxR solos were yet not known. Furthermore, all three *Photorhabdus* species do not contain any *luxI* homologous genes. Therefore, all *Photorhabdus* species found so far do not produce AHLs [5].

Recently, we identified the two homologous LuxR-type proteins PluR and PauR of *P*. *luminescens* and *P*. *asymbiotica*, respectively, detecting each an endogenous signaling molecule used for cell-cell communication. Formerly, both PluR and PauR were classified as LuxR solos, however, since the cognate synthase systems were identified, these receptors are designated as LuxR-type receptors in the following. The LuxR-type receptor PluR of *P*. *luminescens* senses α-pyrones, named photopyrones (PPYs), as signaling molecules at nanomolar concentrations. Moreover, PPYs are produced by the photopyrone synthase PpyS, which is a ketosynthase-like enzyme. *P*. *temperata* possess a PluR-homolog and a PpyS-homolog revealing a similar cell-cell communication via pyrones [6]. Contrarily, *P*. *asymbiotica* comprises neither a LuxI nor a PpyS homolog, thus PauR detects dialkylresorcinols (DARs) and cyclohexanediones (CHDs) as signaling molecules instead of AHLs or PPYs [5]. These signaling compounds are used as well for cell-cell communication and are synthesized by the DarABC operon. Moreover, CHDs are intermediates of the DAR pathway [7]. Upon signal recognition, both LuxR-type receptors activate expression of the cognate *pcfABCDEF* operon leading to cell clumping that contributes to the overall virulence of *Photorhabdus* species. These are highly pathogenic toward insects, whereas *P*. *asymbiotica* is additionally able to colonize and to infect humans. Furthermore, expression of *pcfABCDEF* of either *P*. *luminescens* or *P*. *asymbiotica* in normally harmless *E*. *coli* cells resulted in a pathogenic strain against *Galleria mellonella* larvae. Therefore, the *pcf*-dependent cell clumping and the PpyS/PluR or the DarABC/PauR QS system contribute to the overall toxicity of *Photorhabdus* species towards insect larvae [5, 6].

**Fig 1:**
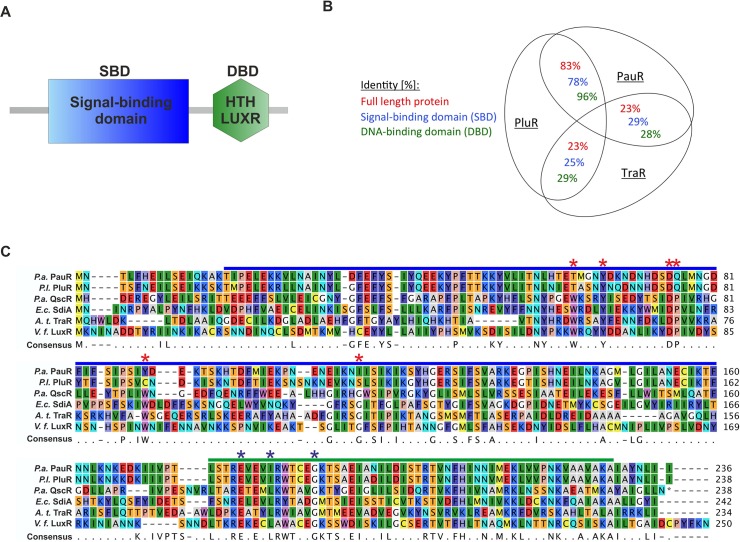
Protein sequence comparison of QS LuxR family members and the non-AHL sensors PluR and PauR. (**A**) Modular domain structure of LuxR-type regulators, with a N-terminal signal-binding domain (SBD) and a C-terminal DNA-binding domain (DBD) [8], containing the helix-turn-helix "HTH LUXR" motif (SMART00421) [25]. (**B**) Comparison of the protein sequence identity of PluR from *P*. *luminescens*, PauR from *P*. *asymbiotica* and TraR from *A*. *tumefaciens*. The identity was compared either of the full-length protein sequence, only the signal-binding domain (SBD) and only the DNA-binding domain (DBD). To calculate identity of the protein sequences the LALIGN software from SIB (Swiss Institute of Bioinformatics) was used [26]. (**C**) Sequence alignment of the protein sequences of PauR from *P*. *asymbiotica* (*P*.*a*.), PluR from *P*. *luminescens* (*P*.*l*.), QscR from *Pseudomonas aeruginosa* (*P*.*a*.), SdiA from *Escherichia coli* (*E*.*c*.), TraR from *Agrobacterium tumefaciens* (*A*.*t*.) and LuxR from *Vibrio fischeri* (*V*.*f*.). The SBD is depicted with a blue bar and the DBD with a green bar. Within the SBD the six conserved amino acids, displaying the WYDPWG-motif of AHL-sensors, are marked with red asterisks and the three conserved amino acids in the DBD are marked with blue asterisks. Amino acids with a consensus of 60–100% are shown, positions with a lower coverage are marked with a dot. The RasMol colouring of the amino acids and the alignment was generated with CLC Mainworkbench 7 (CLC Bio Qiagen, Hilden, Germany).

Certainly, both the LuxR-type receptors PluR and PauR both share the typical domain modularity of QS LuxR proteins, with a N-terminal signal-binding domain (SBD) and a C-terminal helix-turn-helix DNA-binding domain (DBD) [8] (Fig 1A). Upon binding the cognate signaling molecule to the SBD of a LuxR-type regulator, a conformational change is induced, commonly followed by the recognition of target promoter regions by the DBD and transcriptional activation [9]. Furthermore, AHL-binding to AHL-LuxR family proteins is necessary for stability, correct folding [10] or dimerization [11]. LuxR-type proteins share a low protein sequence identity (18%-25%), however, nine highly conserved amino acids are identical in at least 95% of LuxR-type proteins. The SBD harbors six conserved amino acids (W57, Y61, D70, P71, W85 and G113, with respect to TraR, Fig 1C), reflecting a conserved motif for AHL-sensors, which is important for signal-specificity and shaping of the ligand-binding pocket [2]. Three conserved amino acids are located in the C-terminal DBD important for DNA-binding [2] (Fig 1C). The highest conservation of primary structure of several QS LuxR family members is located in the DBD, as its function and mechanism is similar in all LuxR receptors. Whereas the SBD is quite diverse, potentially evolved to an adaptation to specific signaling molecules [12]. The non-AHL sensors PluR and PauR both share a high protein sequence identity among each other compared to typical AHL-LuxR QS family members like TraR from *Agrobacterium tumefaciens* (Fig 1B). Furthermore, PluR and PauR both harbor four substitutions at the similar positions in the conserved WYDPWG-motif of AHL-sensors, displaying a TYDQCS-motif and a TYDQYI-motif, respectively [5]. However, single substitution of the conserved positions Y66 and D75 with alanine in PluR as well as PauR prevented activation by the cognate signaling molecules [5, 6].

In this study we focused on the function of the amino acids of the TYDQCS-motif of PluR and the TYDQYI-motif of PauR in the SBD for binding and/or specificity of the cognate signaling molecule and the functionality of the ligand-binding pocket. Therefore, specific amino acids were replaced one the one hand against A and on the other hand against the respective conserved amino acid at the similar position of QS LuxR family proteins. Furthermore, the conserved motifs of the non-AHL LuxR solos PluR and PauR were restored to possibly sense unrelated signaling molecules to locate the adequate amino acids for AHL- or PPY-sensing.

## Materials and Methods

### Bacteria and growth conditions

The bacterial strains used in this study were *E*. *coli* DH10α [13] and *E*. *coli* LMG194 [14]. The plasmids used in this study are listed in S1 Table and oligonucleotides in S2 Table. *E*. *coli* strains were grown aerobically at 37°C in LB medium [10% (w/v) peptone, 5% (w/v) yeast extract, 10% (w/v) NaCl] or in M63 minimal medium [15] with appropriate antibiotics. Carbenicillin and ampicillin were used at 100 μg/ml and gentamicin was used at 20 μg/ml final concentration. Synthetic C8-HSL (N-3-oxooctanoyl-L-homoserinelactone) was purchased from Sigma-Aldrich and dissolved in methanol. Photopyrone D (PPYD) were isolated and purified from *P*. *luminescens* TT01 supernatant and dissolved in isopropanol [6]. 2,5-dialkylresorcinol (DAR), 2,5-dialkylcyclohexane-1,3-diones (CHDA and CHDB) and isopropylstilbene (IPS) were isolated and purified from *P*. *asymbiotica* PB68.1 supernatant and dissolved in isopropanol [5].

### Generation of plasmids

To monitor the effect of amino acid substitutions in PluR and PauR site-directed mutagenesis was performed to generate *pluR* and *pauR* derivatives. This was achieved with two-step PCR using the appropriate primer pairs and *P*. *luminescens* or *P*. *asymbiotica* genomic DNA, respectively, as template (e.g. PluR_T62W_fwd and PluR_T62W_rev for pBAD24-His-*pluR*-T62W). The overlap PCR was performed using the primers Plu4562-6HisNcoIs and 4562_SalI_rev for *pluR* derivatives and the primers PAU4062-His-NheI_fwd and 4062_SalI_rev for *pauR* derivatives, and the respective DNA-fragment was cloned into plasmid pBAD24 [14] using restriction sites NcoI and SalI or NheI and SalI for *pluR* or *pauR*, respectively. Correct insertion was verified by sequence analyzes using primer pBAD24_Seq_fwd.

### Reporter plasmid assays

To test the specificity of PluR or PauR towards different signaling molecules, *E*. *coli* LMG194 was transformed with the plasmids pBAD24-His-*pluR* and the reporter plasmid pBBR1-*pcfA*_*P*.*l*._-*luxCDABE* or pBAD24-His-*pauR* and the reporter plasmid pBBR1-*pcfA*_*P*.*a*._-*luxCDABE*, respectively. As controls, *E*. *coli* LMG194 was transformed with the plasmids pBAD24-His-*pluR* or pBAD24-His-*pauR* and pBBR1-MCS5-TT-RBS-*lux* (no promoter) or pBAD24 (empty plasmid) and pBBR1-P*pcfA*_*P*.*l*._-*lux* or pBBR1-P*pcfA*_*P*.*a*._-*lux*, respectively. Overnight cultures were grown in M63 minimal medium, adjusted to OD_620_ = 0.05 and then aerobically cultivated in 96-well plates at 37°C. At OD_620_ = 0.1, different signaling molecules were separately added, and the OD_620_ and the luminescence were monitored every hour in a Sunrise plate reader (Tecan, Crailsheim) and a Centro luminometer (Berthold Technologies, Bad Wildbad), respectively. The signaling molecules photopyrone D (PPYD), 2,5-dialkylresorcinol (DAR), 2,5-dialkylcyclohexane-1,3-diones (CHDA and CHDB), isopropylstilbene (IPS) were added in a final concentration of 3.5 nM. The AHL N-3-oxooctanoyl-L-homoserinelactone (C8-HSL) was added in a final concentration of 100 nM. Nomenclature of PPYD is used according to [6] and nomenclature of IPS, CHDA, CHDB and DAR is used according to [5]. As control the same amount of isopropanol or methanol was added. For all strains the relative light unit (RLU) was calculated and subtracted from the respective control strain where only isopropanol or methanol was added. Moreover, highest induction was determined at time point 2 h after addition of substances.

Amino acid substitutions in PluR or PauR might affect the spatial structure of the proteins and influence their functionality to bind the cognate *pcfA* promoter. To quantify the structural influence of amino acid replacements in the signal-binding domain (SBD) of PluR or PauR, the ability of PluR wild type or PauR wild type and different derivatives to activate *pcfA* promoter activity was determined. Therefore, the similar method was used as described above, however 0.1% (w/v) arabinose was added and also derivatives of PluR or PauR were used. For better comparison, the values of PluR wild type or PauR wild type was set as 100% in the Figs 3 and 4.

To quantify the influence of amino acid replacements in the SBD of PluR or PauR on sensing of signaling molecules, the similar plasmid combinations as described above were used, however the distinct signaling molecules PPYD, CHDA, CHDB and DAR were added. The same final concentration of substances was used as described above. For better comparison, the values of PluR wild type or PauR wild type was set to 100% in the Figs 3 and 4.

### Generation of αPluR and αPauR antibodies

To generate specific antibodies against full-length PluR, *E*. *coli* BL21 was transformed with the plasmid pBAD24-His-*pluR*, cultivated at 30°C and expression was induced at OD_600_ = 0.5 with 0.1% (w/v) arabinose. Whole cells were subjected to SDS-PAGE [16], and the amount of 6His-PluR was detected by staining with coomassie solution [40% (v/v) ethanol; 10% (v/v) acetic acid; 0.2% (w/v) coomassie brilliant blue R250] and destained with destaining solution [40% (v/v) ethanol; 10% (v/v) acetate] [17]. The according band with a size of 27.03 kDa was cut and used as an antigen to produce polyclonal antisera and antibodies in two rabbits (Biogenes, Berlin). Furthermore, total IgG of αPluR antibody was purified using Protein-A column (Biogenes, Berlin). Highest specificity of αPluR antibody was given in 3% BSA and a dilution of 1:10.000.

To generate specific antibodies against PauR, polyclonal antibodies were generated in two rabbits against a peptide of PauR (amino acids 62–75: CTMGNYDKNDNHDSD) (Biogenes, Berlin). Total IgG of αPauR antibody was purified using Protein-A column (Biogenes, Berlin). Highest specificity of αPauR antibody was given in 5% milk powder and a dilution of 1:10.000.

### Western Blot analysis

For control of protein production of PluR, PauR and the respectively derivatives, *E*. *coli* strains harboring pBAD-His-*pluR*, pBAD-His-*pauR* or derivatives were cultivated at 37°C in LB. Cells were harvested 2 h after addition of 0.1% (w/v) arabinose and then adjusted to OD_600_ = 1. After SDS-PAGE [16], the proteins were blotted onto a nitrocellulose membrane (Whatman, Germany) in a Mini Trans-Blot cell (Bio-Rad, USA) chamber using a constant power of 100 mA over night. Then, the membranes were incubated with the αPluR antibody (Biogenes, Berlin) or the αPauR antibody (Biogenes, Berlin), as the primary antibody. The α-rabbit alkaline phosphatase-conjugated antibody (Rockland Immunochemicals, Hamburg) was used as the secondary antibody according to the manufacturer’s recommendations. Localization of the secondary antibody was visualized using colorimetric detection of alkaline phosphatase activity with 5-Bromo-4-chloro-3-indolyl phosphate (BCIP) and nitro blue tetrazolium chloride (NBT).

## Results

### PluR and PauR specifically sense their cognate signaling molecule

The SBD of QS LuxR family members mediates specificity towards the respective signaling molecules, on the one hand by shaping the ligand pocket and on the other hand via amino acids that are essential for signal-binding. Primarily, we tested the specificity of PluR and PauR to sense different endogenous or exogenous signaling compounds, like their native signaling molecules or AHLs. For that reason, PluR and PauR were each tested on their ability to sense photopyrone D (PPYD) from *P*. *luminescens*, the *P*. *asymbiotica*-specific signaling molecules dialkylresorcinol (DAR) and its precursors, dialkylcyclohexane-1,3-diones (CHDA and CHDB) and isopropylstilbene (IPS), and the AHL N-3-oxooctanoyl-L-homoserinelactone (C8-HSL). For this purpose the PluR- and PauR-specific reporter plasmid systems, pBAD24-His-*pluR* and pBBR1-*pcfA*_*P*.*l*._-*lux* or pBAD24-His-*pauR* and pBBR1-*pcfA*_*P*.*a*._-*lux*, respectively, were applied.

**Fig 2:**
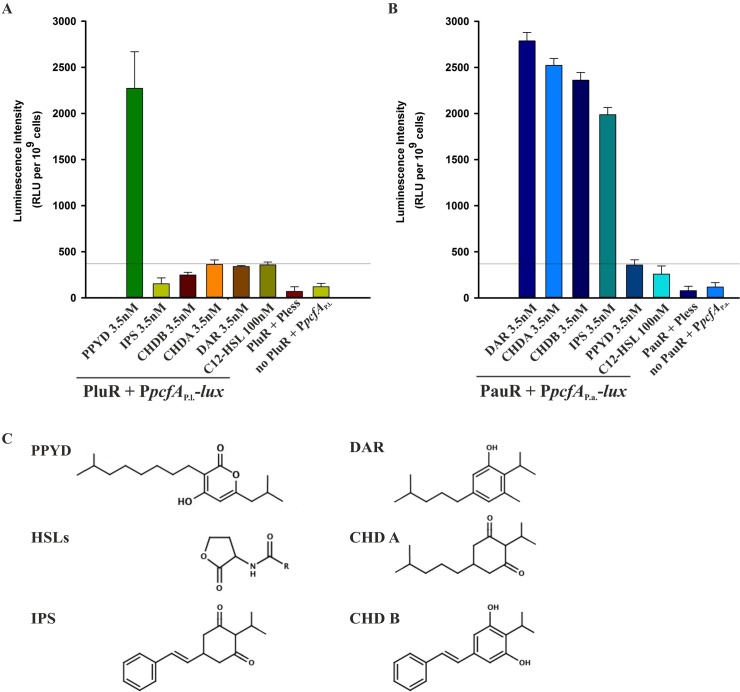
Specificity of PluR and PauR towards different signaling molecules. PluR (**A**) senses its cognate signaling molecule PPYD, whereas unrelated signaling molecules, like C8-HSL or DAR, CHDA, CHDB and IPS, are not sensed. To test the specificity of PluR the reporter system pBAD24-His-*pluR* and pBBR1-*pcfA*_*P*.*l*._-*lux* was used. Similarly, PauR (**B**) specifically senses its native signaling molecules with the highest specificity towards DAR compared to the DAR-precursors, CHDA, CHDB and IPS. The PauR-specific reporter plasmid system composed of pBAD24-His-*pauR* and pBBR1-*pcfA*_*P*.*a*._-*lux* was used. Cells harboring the promoter-less reporter plasmid in combination with each PluR and PauR did not exhibit significant *pcfA* promoter activity. Furthermore, cells harboring the empty pBAD24 plasmid, and therefore no *pluR* or *pauR*, with the respective reporter plasmid as well did not exhibit significant *pcfA* promoter activity. RLUs are shown for 2 h after addition of the depicted signaling molecule. Reference line was set to 370 RLUs to underline the background of the system. RLU, relative light units. (**C**) Comparison of the structures of the signaling molecules used in this study.

To prove suitability of the reporter system, we proved that no luminescence induction occurred either in the presence of one regulator, PluR or PauR, with a promoter-less reporter plasmid or on the other hand in the absence of PluR or PauR with a reporter plasmid containing the *pcfA* promoter of *P*. *luminescens* or of *P*. *asymbiotica*, respectively (Fig 2). Then, PluR as well as PauR were tested for induction of reporter gene activity in the presence of different signaling molecules. PluR senses specifically PPYD with a concentration of 3.5 nM and not unrelated signaling molecules like C8-HSL or the signaling compounds DAR, CHDA, CHDB and IPS from *P*. *asymbiotica* (Fig 2A). Similar to PluR, also PauR showed specificity for it´s native signaling molecules. PauR neither recognizes PPYD nor C8-HSL, but most specifically senses DAR with a concentration as low as of 3.5 nM. Furthermore, PauR senses the DAR precursors CHDA, CHDB and IPS with decreasing sensitivity, tested each with a concentration of 3.5 nM (Fig 2B). Summing up, both LuxR-type regulators PluR and PauR specifically sense the signaling molecule produced by its own species and not to exogenous signaling molecules. None of the two LuxR-type receptors senses C8-HSL, even when added in higher concentrations than their native signaling molecules. Although chemically different, the tested signaling molecules appear structurally somewhat similar in their size and would potentially all fit into the SBD ligand-binding pocket of both PluR and PauR (Fig 2C).

### Specificity of conserved amino acids towards signaling molecule sensing

**Fig 3:**
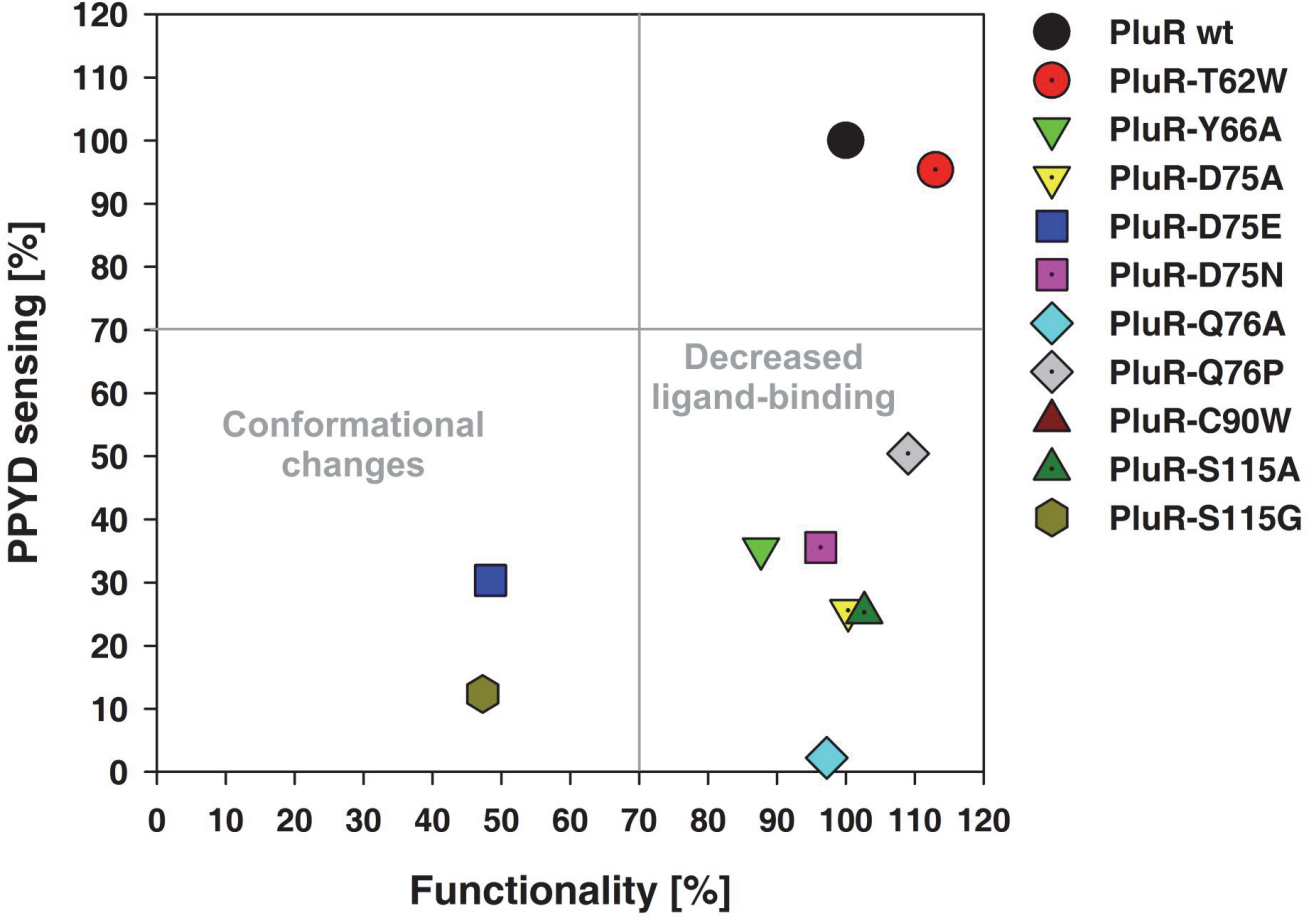
Amino acid replacements within the SBD of PluR caused either functionality or impaired PPYD-sensing. The PluR derivatives D75E and S115G dramatically decreased functionality and hence decreased its ability to bind and activate *pcfA*_P.l._ promoter (lower left quadrant). Replacements within the TYDQCS-motif of PluR decreased the ability of PluR to sense PPYD. The most drastic influence on PPYD-sensing is detectable with the replacement of Y66A, D75A, D75N, Q76A, Q76P and S115A in PluR (lower right quadrant). Only the replacement T62W showed no effect and same induction levels as PluR wild type (upper right quadrant). The activity of the *pcfA*_P.l._ promoter was measured via luminescence as read-out and the depicted values were taken 2 h after addition of 0.1% (w/v) arabinose (lower axis) or 3.5 nM PPYD (left axis) and compared to PluR wild type, which values were set to 100%. To evaluate the different PluR derivatives, a cut-off of 70% was set for each value. RLU (relative light units) values for all PluR derivatives and PluR wild type are depicted in S3 Table.

The SBD of QS LuxR family members harbors six conserved amino acids constituting a conserved WYDPWG-motif for AHL-sensors, which is essential for signal-specificity and shaping of the ligand-binding pocket. PluR and PauR both share only two of the conserved amino acids within AHL-sensors, Y66 and D75, and display a TYDQCS-motif and a TYDQYI-motif at the similar amino acid positions, respectively. Although, the sequence identity between PluR and PauR represents 83% (Fig 1), both sense different signaling molecules. To identify amino acids within each conserved motif essential for signal binding for PluR and PauR, the amino acids within the TYDQCS- and TYDQYI-motif were individually replaced either with alanine or the respective amino acids conserved in AHL-sensors or in PluR. As above, the ability of PluR or PauR and their derivatives were tested to bind and activate the corresponding *pcfA* promoter in presence of the native signaling molecule. To exclude structural influences on the receptor by the amino acid replacements, PluR and PauR wild type and their derivatives were first tested on their general functionality to bind and activate the corresponding *pcfA* promoter. This was tested by activation of *pcfA* promoter activity due to simple overproduction independent of the cognate signaling molecule (Fig 3, lower axis). The amino acid substitutions D75E and S115G in PluR dramatically decreased its ability to activate expression of *pcfA* promoter region to 50% compared to PluR wild type (Fig 3, left lower quadrant). This decreased ability to bind and activate the corresponding *pcfA* promoter, which is possibly caused by conformational defects within one monomer or defects in dimerization. However, all other PluR derivatives that have been tested showed no general functional defects. Furthermore, all tested amino acid replacements in PluR did not influence protein production since protein amounts comparable to the wild type could be detected after overproduction (S1 Fig). Then, the ability of all PluR derivatives was tested for PPYD-sensing. It emerged that the substitutions Y66A, D75A, D75N, Q76A and Q76P in PluR most dramatically impaired signaling molecule sensing (Fig 3, right lower quadrant). The PluR derivative C90W showed a 50% reduced reporter gene activity compared to the wild type. Only the replacement T62W did not affect PPYD-sensing of PluR (Fig 3, upper right quadrant).

**Fig 4:**
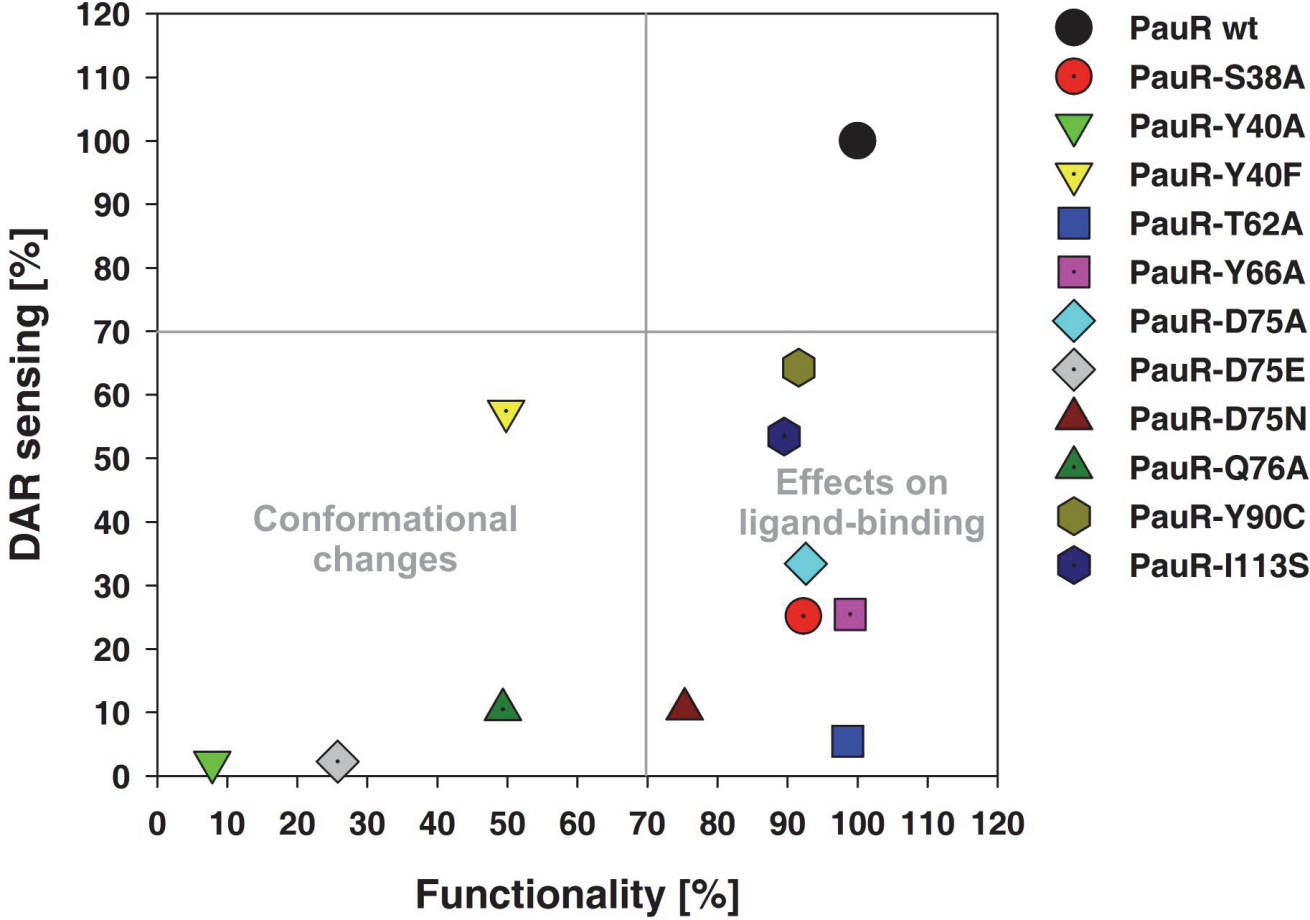
The TYDQYI-motif in the SBD of PauR is essential for the overall functionality of the receptor and DAR-sensing. The most drastic effects on DAR-sensing were gained with the replacement of S38A, T62A, Y66A, D75A and D75N in the SBD of PauR and a decreased effect on DAR-sensing were gained with the replacement of Y90C and I113S in PauR (lower right quadrant). The PauR derivatives Y40A, Y40F, D75E and Q76A dramatically influenced the structure of PauR and decrease its ability to bind and activate *pcfA*_P.a._ promoter (lower left quadrant). The activity of *pcfA*_P.a._ promoter was measured via luminescence as read-out and pictured values were taken 2 h after addition of 0.1% arabinose (lower axis) or 3.5 nM DAR (left axis) and compared to PauR wild type, which values were set to 100% (upper right quadrant). To evaluate the different derivatives, a cut-off of 70% was set for each value. RLU (relative light units) values for all PauR derivatives and PauR wild type are depicted in S4 Table.

Likewise, PauR and its derivatives carrying amino acid substitutions in the TYDQYI-motif were analyzed for their functionality and ability of DAR-sensing. Additionally, the amino acids S38 and Y40 were also analyzed for their impact on DAR-sensing as these have been predicted to be as well involved in DAR-sensing [5]. The amino acid replacements Y40A, Y40F, D75E, D75N and Q76A in PauR dramatically influenced the general functionality of PauR. Thus, over-production of these proteins dramatically reduced *pcfA*_P.a._ promoter activity compared to wild type (Fig 4, lower left quadrant). The most drastic effect appeared when position D75 was replaced with E and when position Y40 was substituted against A (7–25% compared to PauR wild type) (Fig 4, lower left quadrant). Therefore, these amino acids might be important for shaping the SBD and substitutions of these amino acids affect either the monomer structure or dimerization prior to *pcfA* promoter binding. Protein over-production of PauR wild type and all derivatives were comparable revealing that the amino acid substitutions did not effect protein production (S1 Fig). All tested amino acids replacements within the TYDQYI-motif as well as S38A decreased the ability of PauR to sense DAR (Fig 4, lower right quadrant). Replacement of T62A in PauR completely prevented DAR-sensing (5%) without affecting its overall functionality. Furthermore, drastic effects were gained on DAR-sensing with the replacement of S38A, Y66A, D75A and D75N in the SBD of PauR (33–65%) and a decreased effect on DAR-sensing were gained with the replacement of Y90C and I113S in PauR (53–64%) (Fig 4, lower right quadrant).

In both LuxR-type receptors the size of amino acid at position 75 seems to be crucial. When D75 is substituted with a bigger amino acid (D75E) the general functionality of both proteins PluR and PauR is affected (Figs 3 and 4). Whereas charge reversion at position D75N impaired signal-binding and not the general functionality of both PluR and PauR. Furthermore, amino acid Y66 in both proteins is essential for recognizing of the corresponding signaling molecule. Interestingly, also position S38 of PauR is crucial for DAR-sensing (Fig 4). However, this amino acid is not comprised in the conserved TYDQYI-motif but predicted to be involved in DAR-sensing in a DAR-docking model of the SBD of PauR [5]. Overall, all amino acids within the conserved amino acid motif in PluR as well as PauR are important for signal-sensing or functionality, supporting the idea that all these amino acids are located in the respective signal-binding pocket.

**Fig 5:**
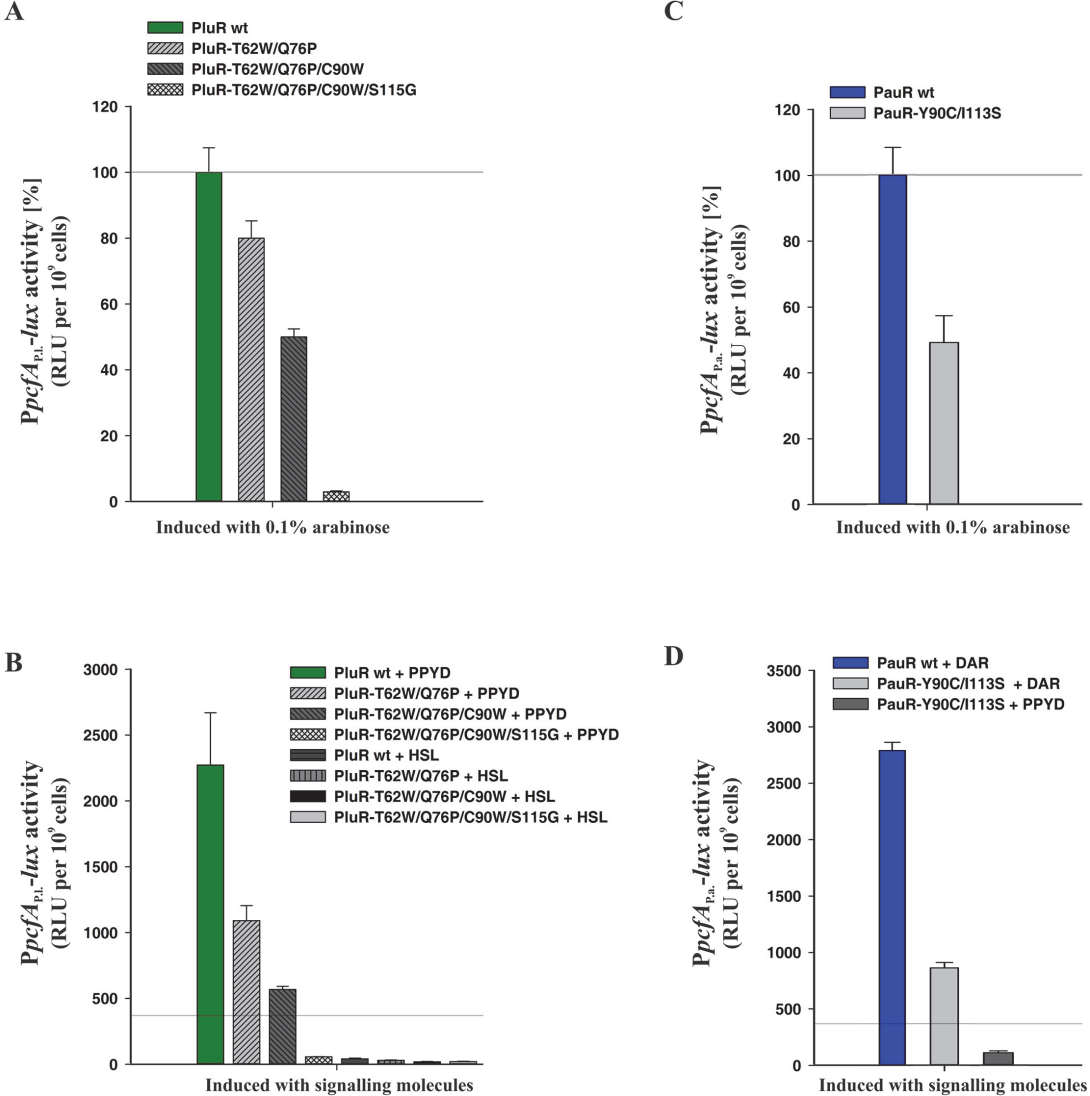
The conserved motifs in the SBD of PluR and PauR are essential but not sufficient for ligand-binding specificity. (**A**) Stepwise replacement of the non-conserved amino acids in PluR to the conserved WYDPWG-motif of AHL-sensors effects the conformation and decreases its ability to activate *pcfA*_P.l._ promoter compared to PluR wild type (wt). The quadruple replacement of PluR-T62W/Q76P/C90W/S115G effects most dramatically the conformation compared to PluR wild type. (**B**) Stepwise replacement of the non-conserved amino acids of PluR decreases its ability to sense its native signaling molecule PPYD, however C8-HSL-sensing could not be gained. **(C)** Replacement of Y90C and I113S in PauR decreased the ability of PauR-Y90C/I113S to activate *pcfA*_P.a._ promoter activity approximately to 50% compared to PauR wild type. (**D**) DAR-sensing was decreased approximately about 70% in the PauR-Y90C/I113S derivative compared to PauR wild type (wt), however, PPYD-sensing could not gained. (**A**) and (**C**): RLUs are shown 2 h after induction and value of PluR or PauR wild type was set to 100% and compared to the respective derivatives. (**B**) and (**D**): The RLU values are depicted 2 h after the addition of the distinct signaling molecules, either 3.5 nM PPYD, 3.5 nM DAR or 100 nM C8-HSL. Reference line was set to 370 RLUs to compare better with Fig 2.

In another approach, we changed the TYDQYI-motif of PluR to the WYDPWG-motif of AHL-sensors to possibly achieve AHL-sensing instead of PPYs. For that reason, we generated quadruple substitutions within the SBD of PluR, resulting in the derivative PluR-T62W/Q76P/C90W/S115G, which was stepwise performed via introducing the next amino acid exchange in PluR-T62W. With successive introduction of more amino acid substitutions within the SBD of PluR, the general functionality of these derivatives is stepwise decreased. However, these derivatives showed comparable protein amounts like wild type (S1 Fig). The quadruple derivative PluR-T62W/Q76P/C90W/S115G strongly impaired the ability to activate the *pcfA*_P.l._ promoter tested by simple over-production of the protein (Fig 5A). Likewise, PPYD-sensing is stepwise impaired with progressive amino acid substitutions in PluR, but AHL-sensing could not be gained (Fig 5B). Furthermore, the reporter gene activities after addition of C8-HSL were lower than the background values of unspecific signaling molecules (compare with Fig 2A). PluR shows the highest protein sequence identity to QscR of *Pseudomonas aeruginosa* of LuxR-type regulators with known crystal structures. The LuxR solo QscR responds to multiple AHLs, like C8-HSL [18], which was therefore used in our study. In conclusion, more amino acids must make up the specificity for AHL-sensing besides the conserved WYDPWG-motif.

Since PluR is very homologous to PauR with a protein sequence identity of 83% (Fig 1), we tried to convert PauR to a PPY-sensor. For that reason, the conserved TYDQYI-motif of PauR was converted to the TYDQCS-motif of PluR to potentially gain PPYD-sensing, though also the general functionality of PauR-Y90C/I113S was affected and reduced to approximately 50% compared to PauR wild type (Fig 5C). However, PauR-Y90C/I113S still had the ability to sense DAR, although this was dramatically reduced about 70% compared to PauR wild type (Fig 5D). Protein amounts of PauR-Y90C/I113S were comparable to PauR wild type (S1 Fig).

Thus, these six conserved amino acids in the SBD, displaying either the WYDPWG-motif of AHL-sensors, the TYDQCS-motif of PluR or the TYDQYI-motif of PauR, are all essential for shaping the specific ligand pocket and ligand-binding, however, these are not sufficient for signal-sensing and-specificity. The solely insertion of the specific amino acid motif for AHL-, PPY- or DAR-sensors into a LuxR-type receptor is therefore not sufficient to convert the signal specificity of the sensor. This reveals that other amino acids in the SBD must also be essential for forming the ligand-binding pocket and for signal perception, although these are not highly conserved.

## Discussion

The two LuxR-type regulators PluR and PauR are both part of a quorum sensing system in *P*. *luminescens* and *P*. *asymbiotica* depending on non-AHL compounds as signals. Both receptors sense different endogenous signaling molecules, PPYs or DARs, respectively, but both activate the expression of the corresponding *pcfABCDEF* operon. Expression of the *pcfABCDEF* operon in turn leads to cell clumping and contributes to the virulence of *Photorhabdus* species [5, 6]. In this study we focused on signal specificity of the two non-AHL sensing LuxR-type receptors PluR and PauR.

PluR and PauR harbor two of the conserved amino acids of the conserved WYDPWG-motif of AHL-sensors, comprising a TYDQCS-motif and a TYDQYI-motif, respectively. However, our studies reveal that these motifs are as important for signal-specificity and conformation as for AHL-sensing of QS LuxR family proteins. Substitution of the conserved amino acid D75 of each PluR and PauR highly decreased the recognition of the cognate signaling molecule. Since D75 of PluR and PauR is deduced to form a hydrogen bond to the hydroxy group attached to the pyrone and the DAR-hydroxy group, respectively [5, 6]. Likewise, in the AHL-sensor TraR this position (D70) is known to be important for binding the amide group of the N-3-oxooctanoyl-L-homoserine lactone [19]. Certainly in PluR and PauR the size and charge of the amino acid at position D75 mediates correct signaling molecule binding as substitution against glutamic acid impaired conformation and substitution against asparagine either affect binding of PPYD or DAR. Docking experiments with PauR and DAR as ligand revealed an arene-arene interaction between T62 and Y66 and the DAR aromatic ring [5]. This was also confirmed by single replacements of both amino acids with alanine, which decreased the ability to sense the ligand (Fig 4). However, in PluR only Y66 was deduced to form an arene-arene interaction with the pyrone ring [6]. Accordingly, substitution of Y66A in PluR showed a dramatically reduced ability to sense PPYD, whereas substitution of T62 against A showed no effect (Fig 3). The appropriate position to Y66 of PluR and PauR is Y61 in AHL-sensors and this amino acid is known to be involved in binding of the acyl chain of the signaling molecule via hydrophobic interactions, e.g. in TraR [19] or LuxR [11]. In general, the six conserved amino acids in the SBD are essential for shaping the ligand pocket and ligand-binding, either in QS LuxR family members binding AHLs or non-AHLs like PluR and PauR. Also the subfamily of LuxR solos of plant-associated bacteria have conserved substitutions in the WYDPWG-motif of AHL-sensors, which are W57M and Y61W (with respect to TraR), however the specific signaling molecules are yet unknown. These substitutions are assumed to allow the binding of plant signal molecules rather than AHLs [2, 20]. Furthermore, several amino acids beside the WYDPWG-motif are known to be involved either in ligand-binding, dimerization or DNA-binding in SdiA from *E*. *coli* [21], TraR from *A*. *tumefaciens* [22] and LuxR from *V*. *fischeri* [23, 24]. This is also true for PauR, in which the position S38 outside of the conserved motif is important for DAR-sensing.

In conclusion, the conserved TYDQCS- and TYDQYI-motifs of PluR and PauR, respectively, are essential but not only sufficient for ligand-binding. Hence, other amino acids of the SBD must also contribute to the signal sensing specificity, although these are not highly conserved. Similarly, QS LuxR family members sensing AHLs contain several important amino acids in the SBD that are important for AHL-binding beside the conserved WYDPWG-motif. Therefore, each QS LuxR-type protein potentially evolved special amino acids to bind its specific signaling molecule to regulate diverse cellular processes. However, PluR and PauR regulate the expression of the cognate *pcfABCDEF* operon leading to cell clumping. Therefore, the question remains why both organisms, *P*. *luminescens* and *P*. *asymbiotica*, use different molecules for this QS-regulated process, which results in a similar phenotype. Possibly, both adapted to their different host, and therefore PPY-signaling might be more appropriate for infection of cold-blooded hosts like insect larvae, and DAR-signaling might be a better choice for infecting endotherm organisms like humans. This idea is underline by the fact that genomic analysis revealed that many human pathogens are putative DAR producers and that these pathogens might also constitute a DAR-dependent QS system, possibly besides an intact AHL QS system [5].

In summary, our studies reveal that specific amino acid motifs in the SBD of LuxR-type receptors are important for signal-sensing, but not alone sufficient for signal-specificity. The replacement of diverse amino acids within the SBD allow LuxR-type receptors to sense diverse families of signaling molecules beside AHLs. The specific amino acid motifs for AHL as well as for PPY and DAR sensors are incomplete to date. Future work has to be performed to identify the complete amino acid motifs in the SBD of LuxR-type receptors, which can then be used for signal prediction of yet un-investigated LuxR solos.

## Supporting Information

S1 Fig Protein production of PluR and PauR and their respective derivatives. For analysis of protein production of PluR and its respective derivatives (**A**) and of PauR and its respective derivatives (**B**), *E*. *coli* strains harboring pBAD-His-*pluR*, pBAD-His-*pauR* or variants were cultivated at 37°C in LB medium. Cells were harvested 2 h after addition of 0.1% (w/v) arabinose, as a control no arabinose was added. The figure shows the immunoblots of 12.5% SDS gels. Antibodies directed against the respective protein were used to detect PluR or PauR. PluR has an estimated size of 27.03 kDa and PauR has an estimated size of 27.14 kDA. The PageRuler prestained protein ladder (Thermo Fischer, Schwerte) was used to estimate protein sizes.
(TIF)

S1 Table Plasmids used in this study. (PDF)

S2 Table Oligonucleotides used in this study. Underlined nucleotides indicate the position of the site-directed mutagenesis.
(PDF)

S3 Table Influence of amino acid substitutions within the SBD of PluR on general functionality and PPYD-sensing. PluR wild type and PluR derivatives were tested for their ability to activate *pcfA*_P.l._ promoter activity controlling the *luxCDABE* operon in the presence of 0.1% (w/v) arabinose or 3.5 nM PPYD. Reporter gene activity was quantified 2 h after addition of 0.1% (w/v) arabinose (functionality [%]) or 3.5 nM PPYD (PPYD-sensing [%]) and compared to PluR wild type, which values were set to 100%. RLU, relative light units. Std, standard deviation of three biological experiments.
(PDF)

S4 Table Influence of amino acid substitutions within the SBD of PauR on general functionality and DAR-sensing. PauR wild type and PauR derivatives were tested for their ability to activate *pcfA*_P.a._ promoter activity controlling the *luxCDABE* operon in the presence of 0.1% (w/v) arabinose or 3.5 nM DAR. Reporter gene activity was quantified 2 h after addition of 0.1% (w/v) arabinose (functionality [%]) or 3.5 nM DAR (DAR-sensing [%]) and compared to PauR wild type, which values were set to 100%. RLU, relative light units.
(PDF)
