# Supplementary figures and images for: Specificity of Signal-Binding via Non-AHL LuxR-Type Receptors
Source: PLoS One. 2015 Apr 29;10(4):e0124093. doi: 10.1371/journal.pone.0124093 (PMC4414361; doi:10.1371/journal.pone.0124093)

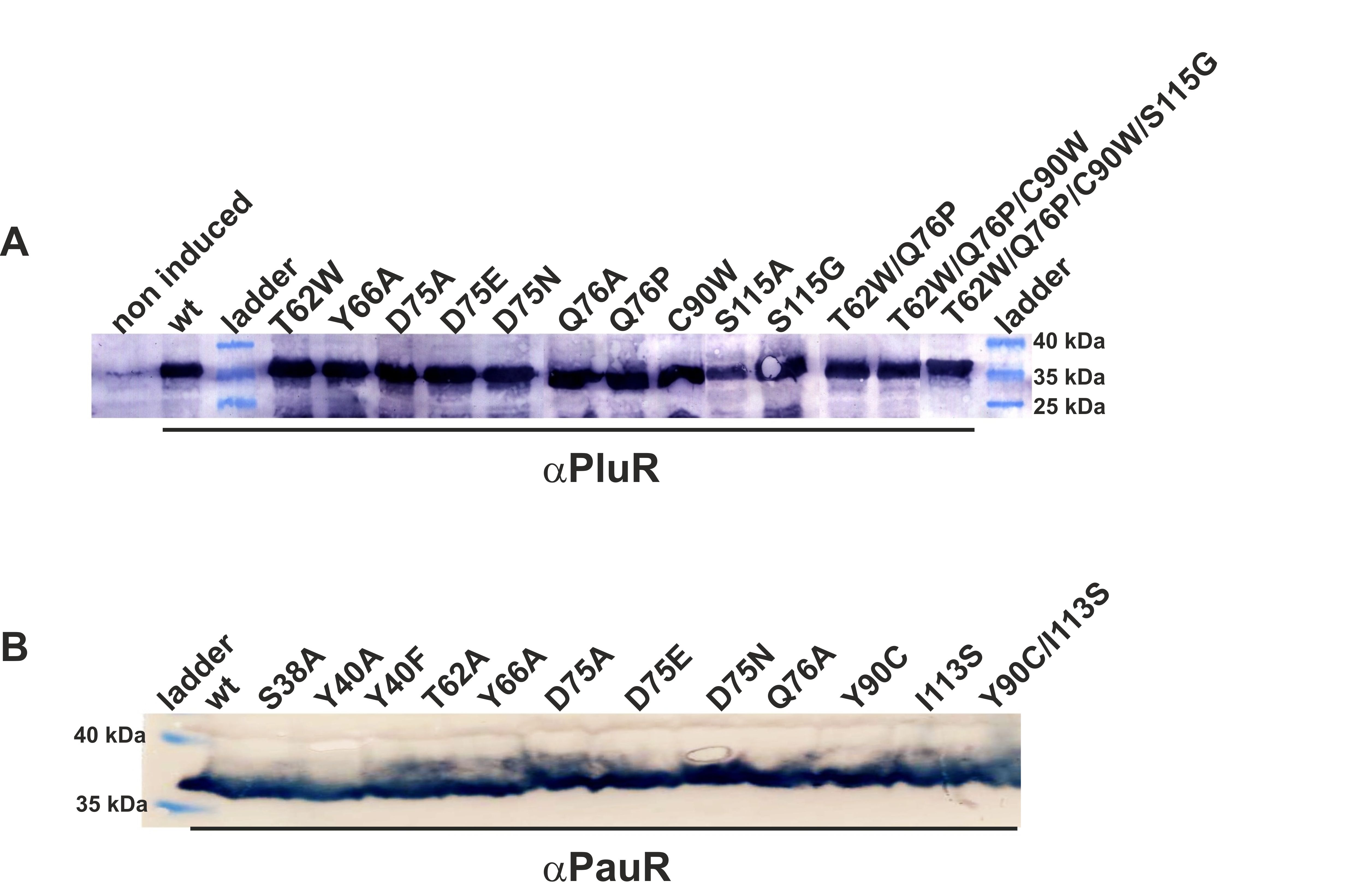

Supplement: S1 Fig — For analysis of protein production of PluR and its respective derivatives (A) and of PauR and its respective derivatives (B), E. coli strains harboring pBAD-His-pluR, pBAD-His-pauR or variants were cultivated at 37°C in LB medium. Cells were harvested 2 h after addition of 0.1% (w/v) arabinose, as a control no arabinose was added. The figure shows the immunoblots of 12.5% SDS gels. Antibodies directed against the respective protein were used to detect PluR or PauR. PluR has an estimated size of 27.03 kDa and PauR has an estimated size of 27.14 kDA. The PageRuler prestained protein ladder (Thermo Fischer, Schwerte) was used to estimate protein sizes. (TIF) [file pone.0124093.s001.tif]
