# Supplementary material for: Specificity of Signal-Binding via Non-AHL LuxR-Type Receptors
Source: PLoS One. 2015 Apr 29;10(4):e0124093. doi: 10.1371/journal.pone.0124093 (PMC4414361; doi:10.1371/journal.pone.0124093)
Supplement: S1 Table — (PDF) [file pone.0124093.s002.pdf]

S1 Table: Plasmids used in this study.

Plasmid	Characteristics	Reference
pBAD24	Expression vector, arabinose inducible promoter, Amp^R	[14]
pBAD24-His-*pluR*	*pluR* (*plu4562*) in pBAD24 with N-terminal His-tag	[6]
pBAD24-His-*pluR*-T62W	Substitution of T62W in *pluR* (*plu4562)* in pBAD24	This study
pBAD24-His-*pluR*-Y66A	Substitution of Y66A in *pluR* (*plu4562)* in pBAD24	[6]
pBAD24-His-*pluR*-D75A	Substitution of D75A in *pluR* (*plu4562)* in pBAD24	[6]
pBAD24-His-*pluR*-D75N	Substitution of D75N in *pluR* (*plu4562)* in pBAD24	This study
pBAD24-His-*pluR*-D75E	Substitution of D75E in *pluR* (*plu4562)* in pBAD24	This study
pBAD24-His-*pluR*-Q76P	Substitution of Q76P in *pluR* (*plu4562)* in pBAD24	This study
pBAD24-His-*pluR*-C90W	Substitution of C90W in *pluR* (*plu4562)* in pBAD24	This study
pBAD24-His-*pluR*-S115G	Substitution of S115G in *pluR* (*plu4562)* in pBAD24	This study
pBAD24-His-*pauR*	*pauR* (*pau_04062*) in pBAD24 with N-terminal His-tag	[5]
pBAD24-His-*pauR*-S38A	Substitution of S38A in *pauR* (*pau_04062)* in pBAD24	This study
pBAD24-His-*pauR*-Y40A	Substitution of Y40A in *pauR* (*pau_04062)* in pBAD24	This study
pBAD24-His-*pauR*-Y40F	Substitution of Y40F in *pauR* (*pau_04062)* in pBAD24	This study
pBAD24-His-*pauR*-T62A	Substitution of T62A in *pauR* (*pau_04062)* in pBAD24	[5]
pBAD24-His-*pauR*-Y66A	Substitution of Y66A in *pauR* (*pau_04062)* in pBAD24	[5]
pBAD24-His-*pauR*-D75A	Substitution of D75A in *pauR* (*pau_04062)* in pBAD24	[5]
pBAD24-His-*pauR*-D75E	Substitution of D75E in *pauR* (*pau_04062)* in pBAD24	This study
pBAD24-His-*pauR*-D75N	Substitution of D75N in *pauR* (*pau_04062)* in pBAD24	This study
pBAD24-His-*pauR*-Q76A	Substitution of Q76A in *pauR* (*pau_04062)* in pBAD24	This study
pBAD24-His-*pauR*-Y90A	Substitution of Y90A in *pauR* (*pau_04062)* in pBAD24	This study
pBAD24-His-*pauR*-1113A	Substitution of I113A in *pauR* (*pau_04062)* in pBAD24	This study
pBBR1-MCS5-TT-RBS-*lux*	*luxCDABE* and terminators lambda T0 rrnB1 T1 cloned into pBBR1-MCS5 for plasmid-based	[25]

	transcriptional fusions; Gm^R	
pBBR1-P*pcfA*$_{P.l.}$-*lux*	*pcfA* (*plu4568*) promoter in pBBR1-MCS5-TT-RBS-*lux*	[6]
pBBR1-P*pcfA*$_{P.a.}$-*lux*	*pcfA* (*pau_04068*) promoter in pBBR1-MCS5-TT-RBS-*lux*	[5]
