# Supplementary material for: Specificity of Signal-Binding via Non-AHL LuxR-Type Receptors
Source: PLoS One. 2015 Apr 29;10(4):e0124093. doi: 10.1371/journal.pone.0124093 (PMC4414361; doi:10.1371/journal.pone.0124093)
Supplement: S2 Table — Underlined nucleotides indicate the position of the site-directed mutagenesis. (PDF) [file pone.0124093.s003.pdf]

S2 Table. Oligonucleotides used in this study. Underlined nucleotides indicate the position of the site-directed mutagenesis.

Oligo	Sequence
Plu4562-6HisNcoIs	5’-GAGGAACCATGGCGCACCACCATCATCACCAT AGGAAAATCTTATGAACACATC-3’
4562_SalI_rev	5’-TAGCCGTCGACTTATATGATTAGATTATATGC-3’
PluR_T62W_fwd	5’-TAAATATAGAATGGGCAAGTAAT-3’
PluR_T62W_rev	5’-ATTACTTGCCCATTCTATATTTA-3’
PluR_Y66A_fwd	5’-GCAAGTAATGCTAATCAAG-3’
PluR_Y66A_rev	5’-CTTGATTAGCATTACTTGC-3’
PluR_D75A_fwd	5’-CATGACAGCGCACAATTAATG-3’
PluR_D75A_rev	5’-CATTAATTGTGCGCTGTCATG-3’
PluR_D75E_fwd	5’-CATGACAGCGAGCAATTAATG-3’
PluR_D75E_rev	5’-CATTAATTGCTCGCTGTCATG-3’
PluR_D75N_fwd	5’-CATGACAGCAACCAATTAATG-3’
PluR_D75N_rev	5’-CATTAATTGGTTGCTGTCATG-3’
PluR_Q76P_fwd	5’-GACAGCGACCCATTAATGAATG-3’
PluR_Q76P_rev	5’-CATTCATTAATGGGTCGCTGTC-3’
PluR_C90W_fwd	5’-CCGTCAGTTTGGAATGATAAA-3’
PluR_C90W_rev	5’-TTTATCATTCCAAACTGACGG-3’
PluR_S115A_fwd	5’-GTCAAAAATGCTCTTTCAA-3’
PluR_S115A_rev	5’-TTGAAAGAGCATTTTTGAC-3’
PluR_S115G_fwd	5’-GTCAAAAATGGTCTTTCAA-3’
PluR_S115G_rev	5’-TTGAAAGACCATTTTTGAC-3’
PAU4062-His-NheI_fwd	5’GAGGAAGCTAGCCGCACCACCATCATCACCATC CCGGGATCTTATGAATACTTTATT-3’
4062_SalI_rev	5′-TAGCCGTCGACTTATATGATTAGATTATATGC-3′
PauR_S38A_fwd	5′-GAATTTTACGCAATTTATCAGG-3’
PauR_S38A_rev	5′-CCTGATAAATTGCGTAAAATTC-3’
PauR_Y40A_fwd	5′-TTACTCAATTGCTCAGGAAG-3’
PauR_Y40A_rev	5′-CTTCCTGAGCAATTGAGTAA-3’
PauR_Y40F_fwd	5′-TTACTCAATTTTCCAGGAAG-3’
PauR_Y40F_rev	5′-CTTCCTGGAAAATTGAGTAA-3’
PauR_D75E_fwd	5′-CATGACAGTGAACAACTAATG-3’
PauR_D75E_rev	5′-CATTAGTTGTTCACTGTCATG-3’
PauR_D75N_fwd	5′-CATGACAGTAATCAACTAATG-3’
PauR_D75N_rev	5′-CATTAGTTGATTACTGTCATG-3’
PauR_Q76A_fwd	5′-CATGACAGTGATGCACTAATG-3’
PauR_Q76A_rev	5′-CATTAGTGCATCACTGTCATG-3’
PauR_Y90A_fwd	5′-CTTCAATTGCTGATGAAAAAAC-3’
PauR_Y90A_rev	5′-GTTTTTTCATCAGCAATTGAAG-3’
PauR_I113A_fwd	5′-GATTAAAAACGCTATTTCC-3’
PauR_I113A_rev	5′-GGAAATAGCGTTTTTAATC-3’
