# Supplementary material for: Specificity of Signal-Binding via Non-AHL LuxR-Type Receptors
Source: PLoS One. 2015 Apr 29;10(4):e0124093. doi: 10.1371/journal.pone.0124093 (PMC4414361; doi:10.1371/journal.pone.0124093)
Supplement: S3 Table — PluR wild type and PluR derivatives were tested for their ability to activate pcfA P.l. promoter activity controlling the luxCDABE operon in the presence of 0.1% (w/v) arabinose or 3.5 nM PPYD. Reporter gene activity was quantified 2 h after addition of 0.1% (w/v) arabinose (functionality [%]) or 3.5 nM PPYD (PPYD-sensing [%]) and compared to PluR wild type, which values were set to 100%. RLU, relative light units. Std, standard deviation of three biological experiments. (PDF) [file pone.0124093.s004.pdf]

S3 Table: Influence of amino acid substitutions within the SBD of PluR on general functionality and PPYD-sensing. PluR wild type and PluR derivatives were tested for their ability to activate *pcfA*$_{P.l.}$ promoter activity controlling the *luxCDABE* operon in the presence of 0.1% (w/v) arabinose or 3.5 nM PPYD. Reporter gene activity was quantified 2 h after addition of 0.1% (w/v) arabinose (functionality [%]) or 3.5 nM PPYD (PPYD-sensing [%]) and compared to PluR wild type, which values were set to 100%. RLU, relative light units. Std, standard deviation of three biological experiments.

	Functionality [%]	std	Sensing of 3.5nM PPYD [%]	std
PluR wt	100.0	± 7.4	100.0	± 7,4
PluR-T62W	113.0	± 6.8	95.4	± 6,8
PluR-Y66A	87.7	± 5.7	35.4	± 5.7
PluR-D75A	100.4	± 11.8	25.5	± 1.8
PluR-D75E	48.4	± 3.2	30.4	± 3.2
PluR-D75N	96.4	± 8.3	35.5	± 8.3
PluR-Q76A	97.2	± 9.1	2.2	± 0.4
PluR-Q76P	109.1	± 5.9	50.4	± 5.9
PluR-C90W	120.7	± 9.3	15.4	± 3.3
PluR-S115A	102.7	± 10.1	25.3	±10.1
PluR-S115G	47.3	± 7.6	12.4	± 4.6
