# Supplementary material for: Specificity of Signal-Binding via Non-AHL LuxR-Type Receptors
Source: PLoS One. 2015 Apr 29;10(4):e0124093. doi: 10.1371/journal.pone.0124093 (PMC4414361; doi:10.1371/journal.pone.0124093)
Supplement: S4 Table — PauR wild type and PauR derivatives were tested for their ability to activate pcfA P.a. promoter activity controlling the luxCDABE operon in the presence of 0.1% (w/v) arabinose or 3.5 nM DAR. Reporter gene activity was quantified 2 h after addition of 0.1% (w/v) arabinose (functionality [%]) or 3.5 nM DAR (DAR-sensing [%]) and compared to PauR wild type, which values were set to 100%. RLU, relative light units. (PDF) [file pone.0124093.s005.pdf]

S4 Table: Influence of amino acid substitutions within the SBD of PauR on general functionality and DAR-sensing. PauR wild type and PauR derivatives were tested for their ability to activate *pcfA*$_{P.a.}$ promoter activity controlling the *luxCDABE* operon in the presence of 0.1% (w/v) arabinose or 3.5 nM DAR. Reporter gene activity was quantified 2 h after addition of 0.1% (w/v) arabinose (functionality [%]) or 3.5 nM DAR (DAR-sensing [%]) and compared to PauR wild type, which values were set to 100%. RLU, relative light units.

	Functionality [%]	std	Sensing of 3.5nM DAR [%]	std
PauR wt	100.0	± 8.3	100.0	± 7.7
PauR-S38A	92.2	± 8.9	25.5	± 2.6
PauR-Y40A	7.8	± 1.6	2.1	± 0.5
PauR-Y40F	49.8	± 5.4	57.4	± 11.1
PauR-T62A	98.6	± 10.5	5.4	± 0.3
PauR-Y66A	98.9	± 10.8	25.4	± 2.8
PauR-D75A	92.6	± 8.1	33.4	± 4.9
PauR-D75E	25.7	± 2.0	2.2	± 0.5
PauR-D75N	75.2	± 4.4	10.5	± 1.9
PauR-Q76A	49.3	± 6.1	10.4	± 1.0
PauR-Y90C	91.6	± 8.1	64.2	± 3.7
PauR-I113S	89.5	± 5.1	53.4	± 7.6
